# Personal recovery and its challenges in forensic mental health: systematic review and thematic synthesis of the qualitative literature

**DOI:** 10.1192/bjo.2021.1068

**Published:** 2021-12-17

**Authors:** Mette Senneseth, Charlotte Pollak, Ragnar Urheim, Caroline Logan, Tom Palmstierna

**Affiliations:** Centre for Research and Education in Forensic Psychiatry, Haukeland University Hospital, Norway; and Department of Welfare and Participation, Western Norway University of Applied Sciences, Norway; Department of Clinical Neuroscience, Centre for Psychiatry Research, Karolinska Institutet, Solna, Sweden; and Stockholm Forensic Psychiatric Clinic, Region Stockholm, Sweden; Centre for Research and Education in Forensic Psychiatry, Haukeland University Hospital, Norway; Centre for Research and Education in Forensic Psychiatry, Haukeland University Hospital, Norway; and Edenfield Centre, Greater Manchester Mental Health NHS Foundation Trust, Prestwich Hospital, UK; Centre for Research and Education in Forensic Psychiatry, Haukeland University Hospital, Norway; Department of Clinical Neuroscience, Centre for Psychiatry Research, Karolinska Institutet, Solna, Sweden; and Stockholm Forensic Psychiatric Clinic, Region Stockholm, Sweden

**Keywords:** Psychiatric nursing, forensic mental health services, psychiatry and law, qualitative research, in-patient treatment

## Abstract

**Background:**

There has been a call for a framework to guide recovery-oriented practices in forensic mental health services.

**Aims:**

This study aims to examine personal recovery and its challenges in forensic mental health settings in relation to the established framework for personal recovery in mental illness: connectedness, hope, identity, meaning and empowerment (CHIME).

**Method:**

This study is an updated and expanded systematic review and thematic synthesis of the qualitative literature. A systematic search of six electronic databases (Web of Science, Medline, PsycINFO, CINAHL, EMBASE and SocIndex) was carried out in January 2019, using the terms [Recover*] AND [Forensic OR Secure] AND [Patient* OR Offend* OR Service User*]. Only studies that included service user's own perceptions and were published from 2014 onward were included in the review. Data were examined with thematic synthesis and subsequently analysed in relation to the CHIME framework.

**Results:**

Twenty-one studies were included in the review. Findings suggest that some adjustments to the original CHIME framework are needed for it to be more relevant to forensic populations, and that an additional recovery process regarding feeling safe and being secure (safety and security) could be added to CHIME, providing the CHIME-Secure framework (CHIME-S). Specific challenges and barriers for forensic recovery were identified and found to represent the opposite of the recovery processes defined by CHIME (e.g. hopelessness).

**Conclusions:**

We present the CHIME-S as a framework for the personal recovery processes of forensic mental health service users. The CHIME-S may guide the recovery-oriented work of forensic mental health services.

A recovery-oriented model of care is well-established as the preferred treatment orientation in mental health services in several European, North American and Australasian countries.^1^ Increasingly, forensic mental health (FMH) services in several countries have started to embrace recovery principles,^2,3^ and the recovery paradigm has widely affected such services in the past decade.^4^ Personal recovery is commonly understood as a deeply personal, unique process and a way of living a satisfying life, notwithstanding the limitations caused by mental disorder.^5^ Within the recovery philosophy, absence of symptoms (clinical recovery) is not the aim; rather, achieving a sense of purpose and mastery in life (personal recovery) is the ultimate goal.^6^ A conceptual framework of personal recovery in mental illness was introduced by Leamy et al,^7^ commonly referred to as the CHIME framework. This framework represents a robust synthesis of people's experiences of personal recovery in mental illness in general psychiatric populations. The acronym CHIME comprises the five recovery processes of connectedness, hope and optimism about the future, identity, meaning in life and empowerment.^7^

In contrast to other mental health service users, FMH service users include mentally ill people whose behaviours represent a considerable risk to themselves and others. Accordingly, they may have very long stays inside restrictive FMH hospitals. Depending on the legal and organisational differences between countries, FMH service users could be sentenced to treatment or referred from general psychiatric hospitals or prisons based on an increased risk of severe violence. The potential conflict between the recovery paradigm and public security features of FMH services has been a subject of debate, and the compatibility between recovery principles and the ethos of secure services has been questioned.^8,9^ For example, it has been argued that service providers may perceive tension between promoting autonomy and choice for patients under conditions of legal coercion.^2,9^ Therefore, it has also been cautioned that applying recovery principles into FMH services may be perceived by staff as merely tokenistic.^10^

Nevertheless, there are strong arguments that the recovery model of care is as important for FMH service users as for other persons with mental illness,^6^ maybe even more important.^11^ Yet, their rehabilitation needs may be more complex.^6^ Efforts have been made in making recovery a reality in FMH services, and previous work has shown that it is possible to implement recovery in a non-tokenistic fashion in forensic settings.^11^ Drennan and Woolridge^11^ propose five key areas for approaches to recovery in FMH services (‘secure recovery’): supporting recovery along the care pathway, the quality of relationships (with staff), risk and safety, meaningful occupation (opportunities for building a ‘life beyond illness’) and peer support. Yet, there is no unifying or established framework for the concept of personal recovery for FMH service users, to guide FMH services.

Two previous reviews have focused on what recovery means for FMH service users, describing their unique recovery processes.^12,13^ The review by Clarke et al^13^ found six superordinate themes defining FMH service users’ perceptions of recovery: connectedness, sense of self, coming to terms with the past, freedom, hope, and health and intervention. The themes ‘connectedness’ and ‘sense of self’ were particularly prevalent.^13^ Shepherd et al^12^ found three key overarching themes: safety and security as a necessary base for the recovery process, the dynamics of hope and social networks in supporting the recovery process, and work on identity as a changing feature in the recovery process. Although some of the themes that emerged in these two reviews may overlap with the recovery processes in mental illness defined by CHIME, findings were not applied to expand or adapt the CHIME framework for use in FMH services. Furthermore, a recent qualitative study of FMH service users’ own views on reducing their risk of serious offending found that the emerging themes fit into personal recovery processes described for general psychiatric populations.^14^ Participants emphasised the importance of trust and creating a context with meaningful relations (connectedness), hope to reach a future goal (hope and optimism about the future) and being given the tools that they needed for their recovery (empowerment). Nevertheless, whether personal recovery processes for FMH service users are fully covered by the CHIME framework remains unclear. Moreover, a recent scoping review of the conceptualisations of personal recovery in mental illness argued that the CHIME framework should be adapted according to client population characteristics.^15^

## Aim

The main aim of the present study is to undertake an updated systematic literature review on personal recovery for FMH service users, and analyse the findings in relation to the CHIME framework. More specifically, this study aims to expand and adapt the CHIME framework to make it suitable for understanding personal recovery in FMH service users, and identify specific challenges and barriers for this client group.

## Method

### Study design

The present study is a systematic review of the existing qualitative literature on experiences of personal recovery in FMH service users. The review applied the thematic synthesis of qualitative research in systematic reviews technique as described by Thomas and Harden.^16^ The review is registered in the International Prospective Register of Systematic Reviews (PROSPERO) register for systematic reviews, under registration number CRD42019128380. The present paper was guided by the Preferred Reporting Items for Systematic Reviews and Meta-Analyses (PRISMA) statement for reporting items for systematic reviews and meta-analyses.^17^

### Eligibility criteria

Papers were included in the review if they applied a qualitative design, were published in peer-reviewed journals, were available in English or Scandinavian languages, and were covering data and findings emerging from the experience of personal recovery in FMH service users.

Exclusion criteria were quantitative papers relying purely on psychometric measures of recovery, perceptions of recovery expressed by others (e.g. staff or family members) and papers not published in peer-reviewed journals (e.g. book chapters, dissertations and the grey literature).

### Search strategy

Six electronic databases (Web of Science, Medline, PsycINFO, CINAHL, EMBASE and SocIndex) were searched with the terms [Recover*] AND [Forensic OR Secure] AND [Patient* OR Offend* OR Service User*]. Building on the two previous reviews by Clarke et al^13^ and Shepherd et al,^12^ the search was subsequently limited to publication years from 2014 onward. The search was conducted on 24 January 2019. An updated search was performed on 7 February 2020. Authors created an alert for the search in Web of Science to identify any new relevant papers throughout 2020.

### Identification and screening of papers

**Fig. 1:**
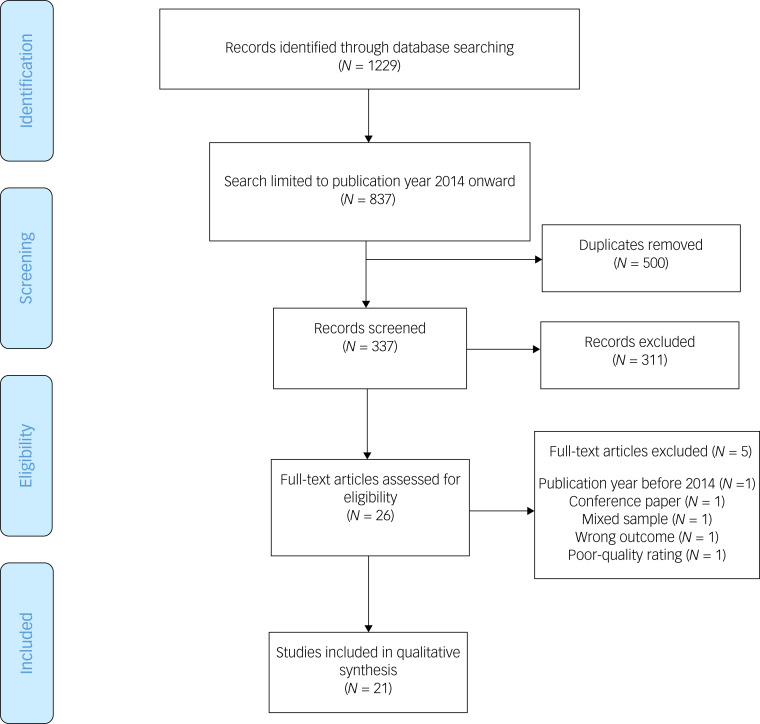
Preferred Reporting Items for Systematic Reviews and Meta-Analyses 2009 flow diagram of the identified and included studies in the present review.

A flow diagram of the identified and included studies in the present review is outlined in Fig. 1. The search identified 1229 papers. After excluding papers with publication years before 2014–2019 (*n* = 392), and removal of duplicates (*n* = 500), a total of 337 papers were screened with the electronic application Rayyan.^18^ Rayyan is a free web and mobile application (available from https://www.rayyan.ai/) that helps systematic review authors perform the initial screening of abstracts and titles.

Four authors screened identified papers individually. Two authors screened half of the papers, and two different authors screened the other half of the papers. In the individual screening process, there was a blinded selection of studies.^18^ If the authors disagreed on the inclusion or exclusion of any paper, Rayyan would demand a second opinion. A second opinion was sought through joint discussions with the research team. All authors assessed the quality of the included papers individually, using the Critical Appraisal Skills Programme (CASP) Qualitative Studies Checklist.^19^ One paper with a poor-quality rating (CASP score < 1) was excluded from the review (Fig. 1).

### Study characteristics

Twenty-one eligible papers were included in the review (Table 1). Of these, 19 were primary studies and two were systematic reviews. The majority of the primary studies were from the UK (seven), followed by Canada (three), Sweden (three), Australia (one), New Zealand (one), China (one), Denmark (one), Finland (one) and Belgium (one). The two systematic reviews covered primary studies from the UK, New Zealand, Canada and Australia. The quality of the included studies were judged to have moderate- to high-quality levels, with CASP ratings ranging from six to ten out of ten (the number of times ‘yes’ was reported in the assessment) (Table 1).

**Table 1:** Study characteristics

Study	Sample	Location and setting	Methodology	Quality assessment (CASP^a^)	Findings represented with themes
Aga et al, 2019^20^	11 interned participants. Mean age 49 years (s.d. 8), aged from 36 to 62 years. Nine men and two women	A variety of internment measures in Belgium	Qualitative in-depth interviews. The data were analysed with an inductive thematic analysis	8	The interviews focused on recovery and elements that indicated a sense of progress in life. Descriptions of recovery resources followed recurrent themes, including clinical, functional, social and personal resources. Participants also reported ambiguous experiences related to features of the judicial trajectory. This was defined as forensic recovery and can be seen as an additional mechanism, besides more established recovery dimensions, that is unique to mentally ill offenders
Askola et al, 2018^21^	Eight forensic psychiatric patients, seven men and one woman, aged from 30 to 50 years. Six in-patients and two out-patients	A Finnish psychiatric hospital with forensic psychiatric patients	Qualitative interviews analysed through narrative analysis	10	The purpose of this study was to describe forensic psychiatric patients’ experiences of and perspectives on forensic psychiatric treatment. Experiences included the quality of relationship with personnel, orienting toward future, turning point, finding hope, peer support, fear of being stigmatised, sticking up for oneself and wanting to show people
Barnao et al, 2015^22^	20 service users, mean age 44 years (s.d. 11.6), aged from 27 to 74 years. Seventeen participants were men and three were female	A New Zealand forensic hospital	Qualitative semi-structured interviews analysed through thematic analysis	10	The analysis identified seven themes that were broadly categorised into those that concerned the rehabilitation context (external) and those that more directly reflected the forensic service user's personal experience (internal). External themes highlighted a person-centred approach, the nature of relationships with staff, consistency of care and awareness of the rehabilitation pathway. Internal themes related to forensic service users’ self-evaluations, agency and coping strategies
Bowser et al, 2018^23^	Eight male participants mean age 35 years, with an age range of 27 years	A medium-secure forensic unit in the South of England	In-depth semi-structured interviews analysed through thematic analysis	9	Emergent themes included: mental health and motivation, restrictive environment, responsibilities and nothing to do
Cherner et al, 2014^24^	20 participants, 18 men and two women. Mean age was 33.6 (s.d. 7.1) years in City A and 33.3 (s.d. 10.7) in City B. Data were available for 18 participants at baseline and 15 participants at 18 months.	TRHP programmes in two cities in Canada	Mixed methods. Client interviews included five open-ended questions about how the program contributed to better functioning and recovery. A grounded theory approach was applied in the analysis	9	Clients described numerous characteristics of community living that contributed to improvements in functioning, such as integration into the community, social contact and newfound independence. Some aspects of TRHP that encouraged recovery included developing new skills and knowledge, staff support and the programming that engaged clients in treatment and recovery-oriented activities
Clarke et al, 2016^13^	A review of 11 qualitative research papers published between 2005 and 2014	The studies were conducted in the UK (*n* = 7), New Zealand (*n* = 2), Canada (*n* = 1) and Australia (*n* = 1)	A systematic review and narrative synthesis of the qualitative literature	10	Two superordinate themes were particularly prevalent: connectedness and a sense of self. It is argued that a focus on increasing opportunities for forensic mental health patients to develop a sense of self and connectedness could help improve recovery
Clarke et al, 2017^25^	Six male patients aged from 32 to 59 years	A low-secure unit comprising three wards (two male, one female) and a two-bed flatlet in Southfield, Southern Health adult forensic service, in the UK	Qualitative semi-structured interviews, analysed using interpretative phenomenological analysis	9	Five superordinate themes were identified: it's a journey, we're vulnerable in here, relationships with staff, loss and hope
Di Lorito et al, 2018^26^	15 participants that were over 50 years old	The region of one NHS Trust in England providing different levels of security: high, medium and low	Qualitative interviews analysed through thematic analysis	9	The interviews were analysed through thematic analysis, which generated seven themes: self-agency, activities, social life, practical matters, recovery, physical health and service improvement. Study findings highlighted the complexity of ageing in secure settings
Glorney et al, 2019^27^	13 male patients, mean age 36 years, aged from 20 to 67 years	A high-secure hospital in South-East England	Qualitative semi-structured interviews analysed through interpretative phenomenological analysis	9	Three superordinate themes were identified: ‘religion and spirituality as providing a framework for recovery’; ‘religion and spirituality as offering key ingredients in the recovery process’; and ‘barriers to recovery through religion/spirituality. The first two themes highlight some of the positive aspects that aid participants’ recovery
Livingston, 2018^28^	18 adults who had received forensic mental health services after being found ‘not criminally responsible on account of mental disorder’. The average age was 35 years, ranging from 27 to 52 years of age	Forensic and general mental health services in Halifax, Canada	Qualitative semi-structured interviews that were analysed with an inductive thematic analytic framework	9	The participants conceptualised success as a dynamic process materialising across six different domains in the context of the forensic mental health system: (a) normal life, (b) independent life, (c) compliant life, (d) healthy life, (e) meaningful life and (f) progressing life
Marklund et al, 2020^29^	11 male participants who had been sentenced for criminal acts. Mean age was 36 years, ranging from 30 to 50 years of age	Four medium-security wards at a forensic psychiatric clinic in northern Sweden	Qualitative semi-structured interviews analysed through qualitative content analysis	10	The analysis resulted in a recurring theme, ‘I know what I need to recover’, and three main categories: ‘a need for meaning in a meagre existence’, ‘a need to be a person in an impersonal context’ and ‘a need for empowerment in a restricted life’
McKenna et al, 2014^1^	Four adult consumers	A 26-bed secure, extended-care facility in Melbourne, Australia	Qualitative one-on-one semi-structured interviews and a general inductive approach was used to analyse the data	8	The seven content domains were (a) a common vision: a journey toward ‘a life worth living’; (b) promoting hope; (c) promoting autonomy and self-determination; (d) meaningful engagement; (e) focusing on strengths; (f) holistic and personalised care; (g) community participation and citizenship and (h) managing risks by taking calculated risks
McKeown et al, 2016^30^	25 male service users	A UK high-secure hospital working to implement recovery practices	Qualitative semi-structured interviews analysed through qualitative content analysis	8	Thematic analysis identified four broad accounts of how recovery was made sense of in the high-secure environment: the importance of meaningful occupation, valuing relationships, recovery journeys and dialogue with the past, and recovery as personal responsibility
Møllerhøj and Stølan, 2018^31^	50 mentally disordered offenders, mean age 40 years, ranging from 19 to 66 years; 44 were male, 6 were female	Either specialised forensic units or in general psychiatry in Denmark	Qualitative semi-structured interviews analysed through qualitative content analysis	6	There are remarkable similarities between the answers, and central points were: the importance of mental health staff acting with respect and empathy in their interaction with patients, improved communication between patients and professionals involved in clinical pathways, responsiveness and shared decision-making when adjusting medical treatment as well as a greater variety of activities offered within in-patient units
Nijdam-Jones et al, 2015^32^	30 participants that were adjudicated ‘not criminally responsible on account of mental disorder’; 24 were male and 6 were female; mean age was 40 years old (s.d. 11.1)	A forensic mental health hospital in British Columbia, Canada	Qualitative semi-structured interviews were analysed with thematic analysis	8	Five themes emerged: involvement in programmes, belief in rules and social norms, attachment to supportive individuals, commitment to work-related activities and concern about indeterminacy of stay.
Olsson et al, 2014^33^	Ten participants (who had decreased their assessed risk for violence on the risk assessment instrument HCR-20, and who were successfully managed on a lower level of security), eight men and two women, aged from 26 to 62 years, with a mean age of 36 years	A maximum security forensic psychiatric clinic in Sweden	Mixed-methods interviews that were analysed with qualitative content analysis	8	Three themes were identified: the high-risk phase: facing intense negative emotions and feelings; the turning point phase: reflecting on and approaching oneself and life in a new way; and the recovery phase: recognising, accepting and maturing
Pollak et al, 2018^14^	Nine in-patients, all sentenced to treatment under the Forensic Mental Care Act with a special discharge trial. Mean age 38 years, aged from 22 to 65 years	The Forensic Psychiatric Clinic of Stockholm County	Qualitative semi-structured individual interviews that were analysed with a structured, qualitative, inductive, data-driven content analysis	10	Four themes emerged: time: opportunity for change; trust: creating a context with meaningful relations; hope: to reach a future goal; and toolbox: tools needed for recovery.
Shepherd et al, 2016^12^	A review of five qualitative studies published between 2000 and 2013	High- and medium-secure units in the UK (*n* = 4) and New Zealand (*n* = 1)	Systematic review and meta-synthesis of qualitative methods studies	9	Three key overarching themes were synthesised: safety and security as a necessary base for the recovery process, the dynamics of hope and social networks in supporting the recovery process, and work on identity as a changing feature in the recovery process
Skinner et al, 2014^34^	Seven service users, mean age 33.7 (range 23–57) years. The majority of the service users were detained under a Section 37/41 Hospital Order and had an index offense of either murder or grievous bodily harm	A motivational programme (the Forward Motion Motivational Group) in a high-secure psychiatric hospital in the UK	Focus groups. The data were analysed with thematic analysis and saliency analysis	9	Five main themes emerged, suggesting that the program had a positive effect on a variety of recovery-related factors, such as confidence, hope, taking control and responsibility, identifying strengths, and improving access to social support
Sustere and Tarpey, 2019^35^	12 male in-patients	Medium-secure units in the UK	Qualitative semi-structured interviews. The data were analysed with thematic analysis	9	Five themes were evident: positive changes, perceived lack of transparency, social isolation, institutionalisation and normality. Patient recovery was promoted through positive risk-taking, the reduction in the use of seclusion and the promotion of meaningful activities that resembled life in the community
Zhong et al, 2019^36^	21 participants with an average age of 45 years, aged from 33 to 62 years, who had lived in the forensic psychiatry hospital for more than 8 years; 19 male and 2 female participants	The Hunan Province Forensic Psychiatry Hospital in China	In-depth semi-structured interviews. The data were analysed with a thematic analysis approach	10	The views and opinions expressed by long-stay patients showed that psychological distress is prevailing in forensic psychiatric hospital. Participants’ perceptions clustered into seven themes: hopelessness, loneliness, worthlessness, low mood, sleep, disturbances, lack of freedom and lack of mental health intervention

CASP, Critical Appraisal Skills Programme; TRHP, Transitional Rehabilitation Housing Pilot, HCR-20, The Historical, Clinical and Risk Management.

a CASP quality assessment, the number of times ‘yes’ was reported in the assessment (Range 0–10).

The total sample of participants (FMH service users) in the primary studies was 298, of which the majority were in-patients (*n* = 274) from different levels of security: high, medium and low.

### Data extraction and analysis

The method of data extraction and data analysis followed the three steps of the thematic synthesis described by Thomas and Harden.^16^ The main aim of the thematic synthesis is to preserve an explicit and transparent link between conclusions and the text of primary studies.^16^ The three stages involve the free line-by-line coding of the findings of primary studies, followed by the organisation of these free codes into related areas to construct descriptive themes and, finally, the development of analytical themes. Data extraction started on 17 September 2019. The result sections of the 19 primary studies and the two systematic reviews were coded line by line, by the four authors individually, according to the first stage in the thematic synthesis.^16^ The codes were entered in an Microsoft 365 Excel for Windows worksheet. All associated sentences were entered together with the codes in a different column, to keep track of content, meaning and quote. The systematic reviews were handled the same way as the primary studies, to be sure to include and retain relevant themes that were reported in studies before 2014.

Data analysis started on 19 February 2020. All authors worked with the thematic synthesis in joint meetings where at least three authors were present at all times. According to the second stage in the thematic synthesis, authors organised these free codes and constructed them into descriptive themes. The descriptive themes were subsequently analysed within the established CHIME framework for personal recovery in mental illness. After checks of content and meaning, all descriptive themes that would clearly fit in CHIME categories or subcategories were allocated there. When additional subcategories or second-order subcategories were needed to account for the content and meaning of the new descriptive themes, they were added to the original CHIME categories.^7^ When descriptive themes were negatively loaded, they were marked with a minus in the worksheet. If the majority of codes within a category were negatively loaded, these were analysed separately as ‘challenges and barriers’. These represented factors that may be a challenge for personal recovery in FMH services or actual hindrances that ultimately may limit the recovery processes.

Themes that did not clearly fit into any existing CHIME category or subcategory were further analysed and developed into analytical themes, according to the third stage of the thematic synthesis. The new analytical themes were suggested as new main categories. In line with the experience of Thomas and Harden, the stages overlapped to some degree.^16^ A final check of codes found to fit into the new main categories and subcategories was performed by two of the authors, and relevant quotations were extracted from the papers to illustrate the new categories. The method applied here has strong similarities with the ‘best-fit’ analysis,^37^ which is a framework synthesis approach to the qualitative systematic review. The best-fit analysis applies the principles of the standard thematic analysis. However, the present study has applied the thematic synthesis approach, which includes the more detailed line-by-line coding of the data.

All authors have clinical experience in forensic psychiatry. To aid reflexivity, the research team was helped by research notes throughout the analysis process. Through discussions in joint meetings, authors worked systematically with the notes to focus on the service user perspective; namely, what is personal recovery for the FMH service users, and not what is good for the services or what services think is recovery for service users. Here, authors needed to interpret findings from a consumer perspective and not from their own judgements, being true to the statements from service users in the original papers. Consensus was made from these discussions.

### Ethical considerations

Service user consent was not required for this systematic review of the already published literature. However, the populations of the studies reviewed were vulnerable groups that required additional protection. The CASP quality checks revealed weakness in the reporting of the relationships between researchers and participants. It is considered to be a higher quality of reporting of a qualitative paper if the relationship between the researcher and participants has been adequately considered.^19^ However this was not the case in 12 out of 21 studies. This issue should be addressed in future research within FMH services.

## Results

The data comprised 1760 line-by-line codes derived from the results sections of 21 included papers. The majority of codes (74%) were related to personal recovery processes for FMH service users. Most findings corresponded with the existing five main recovery processes in the CHIME framework.^7^ However, one new main recovery process category emerged from the data. This new category was labelled ‘safety and security’ (‘secure’), and consisted of issues related to feeling safe and being secure, including safe care pathways and self-management of risk. The original category of connectedness was expanded to include ‘staying connected and being part of a ward community for a long time’. Hope and optimism about the future was expanded to include ‘looking back and looking forward’. Identity was expanded to include ‘identity work: coming to terms with trauma and past offences’. Meaning in life was expanded to include ‘meaningful use of time on the ward and preparing for a meaningful life outside’. Empowerment was expanded to include ‘empowering collaboration within a frame of restrictions’. Four new subcategories and 21 new second-order subcategories emerged from the analysis, which were added to the original CHIME framework to define the forensic version, making it the CHIME-Secure (CHIME-S) (Table 2) (for the full version of the table, see supplementary material available at https://doi.org/10.1192/bjo.2021.1068).

**Table 2:** CHIME-Secure: recovery processes for forensic mental health service users^a^

	Number of codes	Number of studies represented
1 Connectedness	**353**	**21**
1.1 Peer support and support groups	**21**	**9**
**1.1.3 Supporting others**	8	3
1.2 Relationships	**36**	**13**
**1.2.4 Repairing relationships**	2	2
1.3 Support from others	**111**	**18**
1.4 Being a part of the community	**36**	**11**
**1.5 Relationships with staff**	**83**	**15**
**1.5.1 Respect**	15	8
**1.5.2 Trust**	10	6
**1.5.3 Being seen as an individual**	18	7
**1.5.4 Staff spending time with patient**	6	2
**1.5.5 Quality of therapeutic relationships**	30	12
**1.6 Being a part of a ward community**	**66**	**14**
**1.6.1 Supportive environment**	28	8
**1.6.2 Being supported despite drawbacks**	4	2
**1.6.3 Support from structure and activities on the ward**	34	12
2 Hope and optimism about the future	**140**	**21**
2.1 Belief in possibility of recovery	**66**	**16**
**2.1.1 Looking back and looking forward**	5	4
2.2 Motivation to change	**17**	**8**
2.3 Hope-inspiring relationships	**11**	**9**
2.4 Positive thinking and valuing success	**22**	**10**
2.5 Having dreams and aspirations	**16**	**12**
**2.5.1 Hope for an ordinary life**	8	6
3 Identity	**174**	**19**^b^
3.1 Dimensions of identity	**6**	**3**
3.2 Rebuilding/redefining positive sense of self	**144**	**16**
**3.2.4 Working with one's identity**	16	8
**3.2.5 Coming to terms with past offences**	55	11
**3.2.6 Coming to terms with trauma and having been victimised**	5	2
3.3 Overcoming stigma (total)	**24**	**11**
4 Meaning in life	**225**	**20**^c^
4.1 Meaning of mental illness experiences	**31**	**9**
4.2 Spirituality (including development of spirituality)	**15**	**2**
4.3 Quality of life	**85**	**17**
4.4 Meaningful social and life goals	**11**	**7**
4.5 Meaningful life and social roles	**12**	**6**
4.6 Rebuilding of life	**43**	**13**
**4.6.3 Preparing for life outside forensic hospital**	6	4
**4.7 Meaningful use of time**	**28**	**10**
**4.7.1 Spending time outside the ward**	11	5
**4.7.2 Active use of time**	15	7
5 Empowerment	**321**	**20**^d^
5.1 Personal responsibility	**83**	**19**
5.2 Control over life	**208**	**20**
**5.2.5 Clear goals**	13	5
5.3 Focusing upon strengths	13	8
**5.4 Mutual collaboration**	**17**	**7**
**5.4.1 Common view of means and goals**	9	4
**5.4.2 Cooperation and involvement**	8	5
**6 Safety and security**	**94**	**19**^e^
**6.1 Helpful restrictions**	**21**	**4**
**6.2 Feeling safe and protected on the ward**	**29**	**9**
**6.3 Self-management of risk for violence and/or relapse in crime**	**40**	**13**
**6.3.1 Taking responsibility for own actions**	17	11
**6.3.2 Health-maintaining and risk-reducing strategies**	23	7
**6.4 Safe plans for the future**	**4**	**3**

a Categories shown in bold are additions to the original connectedness, hope, identity, meaning and empowerment (CHIME) framework.^7^ Total numbers are given in bold.

b All studies except McKenna et al^1^ and Marklund et al^29^ are represented.

c All studies except Barnao et al^22^ are represented.

d All studies except Shepherd et al^12^ are represented.

e All studies except Bowser et al^23^ and Zhong et al^36^ are represented.

Barriers and challenges to personal recovery for FMH service users constituted 26% of all codes (Table 3). Eight papers also described characteristics of the specific forensic recovery journey.^1,14,21,25,28,30,31,33^

**Table 3:** Specific challenges and barriers in forensic recovery

	Number of codes	Number of studies
Challenges and barriers to personal recovery for forensic mental health service users	**453**	**19**
Disconnectedness	**93**	**14**
Loneliness	12	3
Not being respected	37	8
Not having trust in staff	24	8
Lack of social interaction because of restrictions	8	4
Dilemma of disclosure	5	3
Hopelessness	**17**	**10**
Negative identity experience – stigma as offender	**34**	**9**
Lack of meaning	**58**	**8**
Boredom	36	7
Waste of time	19	6
Disempowerment	**251**	**19**
Uncertainty of indefinite time of internment	32	11
Lack of clarity in treatment and plans	17	7
Being subjected to disempowerment	81	13
Limited by rules and restrictions	39	13
Adapt to rules and care with resignation	7	4
Lack of collaboration	54	10
Feeding the beast	15	7
Loss of freedom	6	2

Main categories and total numbers within categories are given in bold.

### Key recovery processes for FMH service users

The findings related to each of the six key recovery processes that define the CHIME-S framework are outlined below.

#### Connectedness: staying connected and being part of a ward community for a long time

Most of the findings were related to the key recovery process of connectedness, which was represented in all the reviewed papers. A major part of the findings regarding connectedness was concerned with service users being a part of the ward community and their relationships with staff. The quality of relationships with staff and the ward environment played an important role in forensic service users’ recovery.^13,14,21,22,25–27,30,33,35^ The quality of relationships with staff were viewed as essential.^25,29,30,32^ One service user stated:

‘It's all about the relationships with staff. I wouldn't even have started my recovery if I hadn't started to trust some staff, and this wouldn't have happened without them being ok with me. Yes, I'd say the relationships is the important bit. Nothing is going to happen without good relationships.’^30^ (p. 237).

Another service user expressed: ‘I have applied to get leave and the staff have supported that. It's important to know that my clinical team are all behind me’^26^ (p. 943). Features that characterised good-quality relationships with staff were showing respect toward service users, having a genuine interest in them and their well-being, being empathic and being flexible in their interaction with them.^21,25,26,30,31^ One service user stated: ‘(…) A good personal nurse can always be bothered with you, whenever I was depressed he was there to support me’^21^ (p. 5). Furthermore, service users valued staff who spent time with them, were helpful and worked collaboratively with them to support change.^20,22,29^

Service users emphasised the importance of having trust in staff, feeling encouraged and supported by them, and not feeling looked down on by them.^1,20,26,29,30,32^ In fact, it was essential to forensic service users on their recovery journeys simply to be treated as individuals and human beings.^20,22,31^ One patient stated: *‘…* although we are people with mental problems, we are still human beings with emotions and feelings’^31^ (p. 595). Furthermore, many service users accepted and hoped for long-term support from professionals as part of their enduring recovery journey.^1,31,33^

Environments that were recovery-promoting were characterised by being calm, supportive and safe, and a place to socialise and to be supported within despite drawbacks.^20,26,29,33^ Some felt that their recovery was positively supported by structures, routines and the daily activities on the ward.^14,20,33^

Forensic service users described relationships with family, friends, peers and staff as essential for their recovery.^12,13,20,28,29,32^ However, some needed help from professionals in re-establishing and repairing existing relationships,^24,30,31^ which may be specific for the forensic service users and relevant to their history of offending behaviour. Two papers also highlighted forensic service users’ desire to help and be supportive of others.^20,27^

#### Hope and optimism about the future: looking back and looking forward

Developing hope for a good life in the future and the belief in the possibility of recovery was viewed as an essential recovery process in all 21 papers. FMH service users found that supportive relationships with family, friends and staff fostered hope,^12,13,35^ as did clear goals for their treatment,^34^ and the experience of interventions and procedures that supported personal autonomy.^12^

Many service users described hope for an ordinary life,^28,29,31^ a ‘normal life’ worth living.^1^ They aspired to having a safe place to live, family and friends, and paid work.^28,29,31^ Although they aspired to independent living, several service users recognised a need for long-term support from professionals as well as life-long medication.^1,31,33^

Several papers described how time was viewed as an opportunity for change.^21,30–33^ Some FMH service users had experienced a turning point in their recovery trajectory.^14,21,30,33^ This turning point was characterised by looking back on the past, and then looking forward to the future with a sense of hope.^30^ One service user stated: ‘Seeing the progress and being able to move forward—keeps that hope going’^28^ (p. 222). Olsson et al^33^ found that being able to weigh pros and cons of the past and the present and look back on their life gave FMH service users strength, because the future looked brighter than the past and this helped to strengthen the turning point process. In the study by Clarke et al^25^ one service user stated: ‘So you get to leave all the bad behind you and be someone new. That's something to look forward to’ (p. 69). Valuing success and experiencing an understanding of their illness and past, were additional key features of this turning point, in addition to developing insight^21^ and making a conscious decision to change.^30^ A service user in the study by McKeown et al^30^ said ‘It's all about me getting better. I see the point of the medication now. I've been really ill and now I'm moving on’ (p. 237). Another service user stated: ‘(…) I've had a lot of time to think about what I've done in the past. I've had to make an effort and make a conscious decision to change’ (25, p. 237).

#### Identity: identity work, coming to terms with trauma and past offences

The identity category was represented in 19 of the 21 reviewed papers (Table 2). Analyses revealed three new second-order subcategories related to rebuilding and redefining a positive sense of self: ‘working with one's identity’, ‘coming to terms with past offences’ and ‘coming to terms with trauma and having been victimised’. Forensic service users described how working with one's identity had been challenging as well as beneficial for their recovery.^25^ They viewed coming to terms with their past offending as essential, as well as coming to terms with the experience of trauma and of having been victimised themselves.^13,25,27^ Some stated the need for to accept the past,^34^ to forgive oneself^27^ and to leave the past behind.^20,25^ Others described how processing their crime in therapy was a major part of moving on.^14^ Several participants in the study by Olsson et al stated that they suddenly felt a need to change their way of being.^33^ Working with their identities, service users described reflecting on and approaching themselves and life in a new way.^33^ Self-reflection and honesty toward themselves were mentioned as additional key elements in their identity work.^20,24^ Others stressed the importance of relationships with staff in developing their new identities when building up ‘this whole new person’^25^ (p. 65). Service users described how this process of recovery was hard, painful and exhausting work.^14,25,30^ One participant in the study by Askola et al^21^ stated:

‘It was pretty difficult. It was difficult to process, especially at the beginning. It was difficult to accept having done such a thing or that it should still somehow be worked through and got over, that this has happened’ (p. 5).

Service users reported that they had had a lot of time to think about what they had done in the past.^30^ Some service users described feelings of guilt, shame and remorse,^36^ and they expressed a need for encouragement from staff to work through this particular recovery process.^21,32^ One service user expressed:

*‘*My index offence was so terrible. I wouldn't have been able to go on much longer personally without their [staff] support … Even going forward … they're reminding me that … I never would have done what I did had I not gotten mentally ill. Their support [in] so many different ways has benefitted me’^32^ (p. 163).

Furthermore, one paper described how the role of the victim was important for moving forward, either through the fact that the victim survived or through the restorative effect of contact with the victim (on the victim's terms) or by helping them.^20^

#### Meaning in life: meaningful use of time on the ward and preparing for a meaningful life outside’

All reviewed papers but one documented the service users’ need to experience meaning in life as well as an adequate quality of life while being an in-patient, in addition to an active and meaningful use of time (Table 2). Several activities were seen as beneficial for enhancing perceived quality of life, such as the opportunity to participate in recreational activities,^23,24^ paid work or work-related activities,^20,23,28,30,32^ and leisure activities.^20,26^ Service users appreciated spending the day in a purposeful way and acknowledged that keeping busy was good and important to maintaining mental health.^23,30,32,33^ One service user stated: ‘I've got quite a busy schedule here and if I didn't, I know that keeps me ticking over. I need that to keep me going. Being busy is good (…)’^30^ (p. 236). An active use of time was viewed as essential for reducing boredom and improving mood,^23,27,32^ as well as for reducing the risk of aggression and violence.^30,31^

Several papers documented a call for more activities^29,31^ in addition to opportunities to experience joy.^31^ Activities offered peace of mind and distraction,^20,23,26^ but also provided a way to pass time, which was thought to be helpful.^20^ However, service users needed to feel that the activities were meaningful to engage with them and to sustain their interest: ‘If they were too easy, too difficult, or repetitive then they were perceived as boring’^23^ (p. 39). Being allowed leave was considered as particularly helpful as it gave a taste of freedom and hope for the future.^13,25,35^ Spending time outside the wards doing outdoor activities was also highly valued,^20,24,25,31^ giving service users a sense of freedom and a glimpse of normality.

Furthermore, several papers emphasised service users’ needs regarding preparing for life outside FMH services.^1,29,32,34,35^ Service users wanted the content of care to support them in preparing for life outside of FMH hospital,^29^ and to help them develop skills that they needed to achieve and maintain a good life.^32,35^ They embraced education and training programmes to help them develop occupational skills.^32^ These skills included skills to cope with difficulties and stigma,^32^ support and skills at maintaining social relationships,^36^ independent living skills, learning skills in decision-making and relapse prevention.^24^ Developing new skills came with additional beneficial effects including increased self-esteem and self-worth,^13^ a sense of achievement,^32^ as well as feeling more prepared for discharge.^35^ Furthermore, relevant training was viewed as a step toward successful community reintegration.^32^ Service users linked having a reason to get up every morning and having much to lose, with a lower risk of reoffending.^28^

#### Empowerment: empowering collaboration within a frame of restrictions

Codes that were related to the process of empowerment constituted the second largest amount of the findings, followed by that of connectedness, and were reported by 20 papers (Table 2). The findings highlighted the needs of FMH service users for a sense of empowerment in a restricted life.^29^ Service users called for clear goals in their care,^24,25,33,34^ and felt themselves to be inadequately informed about the objectives of their care and treatment.^21,35,36^ Although all papers stressed the need for a mutual collaboration in forensic care, many service users experienced a lack of collaboration and involvement.^1,14,22,25,33^ One paper revealed that service users lacked knowledge about the legal processes of discharge, and were told to stay calm and follow the rules.^36^ For those who had experienced some degree of collaboration and involvement in the delivery of their care, this was highly valued and seen as an important element of their recovery. Service users called for a mutual collaboration with health professionals about their care.^22,25^ Mutual collaboration included sharing common views of interventions and goals, experiencing cooperation with health professionals, ‘singing from the same hymn book’ and being involved in their own care.^13,29^

Increased knowledge about illness and treatment as well as legal procedures was helpful when presented with choices. Conversely, a lack of information was seen as unlikely to help service users to become more independent and promote their recovery.^35^ Furthermore, if the reasons for decisions were explained or if the service users were invited to express their opinions about them, then decisions were easier to accept.^30^ Regaining freedom, independence and autonomy were the ultimate goals of many FMH service users. Marklund et al^29^ concluded that service users ‘… dreamed of living on their own, starting work, having a meaningful everyday life, and being free’ (p. 4). Furthermore, to have access to treatment programmes and medication was viewed as important to being able to recover.^32^

#### Safety and security: feeling safe and being secure, experiencing safe care pathways and self-management of risk

A prevalent finding in almost all papers was that service users needed to feel safe and secure, which meant being protected from hostile people and environments and the active practice of self-management of risk. The majority of findings in this new main category were related to self-management of risk of violence and/or relapse to criminality. These findings were present in twelve of the reviewed papers. Self-management of risk comprised ‘taking responsibility for own actions’ and ‘health-maintaining and risk-reducing strategies’. Taking responsibility for own actions included ending their denial of their mental health difficulties and their offending behaviour,^34^ and staying out of trouble.^1,22^ Skinner et al quoted one service user who stated: ‘You have to understand that you're the one that committed that action, so it helped me become more responsible’^34^ (p. 94). In the study by Askola et al,^21^ one patient stated: ‘It is important to be honest with yourself. Ultimately, you only screw up yourself. I have the rest of my life involved, and it motivates me’. The importance of motivation for taking responsibility for own actions was also highlighted by Olsson et al.^33^ They describe how FMH service users, who had earned trust of treating physicians and got outside privileges, would be unwilling to take risks that might lead them to lose what they already had achieved.

The significance of being able to staying out of trouble was highlighted by several FMH service users.^1,28^ In the study by Livingston,^28^ one service user said: ‘You have to be able to stay away from problems with the law’ (p. 217). Similarly, a service user in the study by McKenna et al^1^ stated that recovery is to: ‘… keep out of jail, keep off the street, keep out of hospitals, and not relapse (…)’ (p. 65). Sustere and Tarpey^35^ cited a service user who stated that ‘recovery means … being able to manage your problems and living in the community without re-offending and so looking after yourself’ (p. 621). Several health-maintaining and risk-reducing strategies were described to be helpful for becoming more confident in coping with difficulties and risks. Health-maintaining strategies included taking medication,^1,14,20,21,28–31,33^ staying away from drugs and alcohol,^28,31^ and developing new health-promoting skills such as relapse-prevention skills, coping strategies, medication management and social skills.^24^ Many service users found participating in different programmes as particularly helpful in developing new skills.^28,34,35^ Some felt that religion helped them to reduce their risks as it provided rules for them to live by: ‘The Ten Commandments tell me I must not steal, must not kill, mustn't do a lot of different things, and if follow those, then I won't, I won't relapse, ‘cos a relapse for me would constitute violence’^27^ (p. 193).

Several papers described the need to feel safe and protected on the ward to support recovery.^12,13^ Service users were vulnerable to being victims of intimidation and violence on wards,^25^ and they needed protection from violence from fellow service users as well as from a hostile public.^13^ The presence of staff contributed to the feeling of safety and being protected,^31^ and quieter wards felt safer and nurtured the motivation to change.^33^ Safe care pathways included safe transitions as well as safe plans for the future. Service users wanted to know that there was a plan for their independent living and a vision of transition to out-patient care,^29^ as well as, eventually, a safe place to live.^31^

Four papers highlighted the dual role of restrictions, showing that although restrictions could often be a barrier to personal recovery, they could also be helpful.^13,14,20,32^ One example was a prohibition on alcohol consumption, which was found helpful by some.^20^

Another example was the prevention of crime through internment,^14^ as well as the prevention of their injury or death: ‘If I hadn't been interned, I would be six feet under by now (…)’^20^ (p. 928). Moreover, one paper described how adherence to rules was seen as a way of showing respect to those who helped them as well as respect for the law.^32^

### Specific challenges and barriers for personal recovery for FMH service users

Several challenges and barriers for personal recovery emerged from the data (Table 3). Although mentioned as important for recovery, and being thematically in line with the recovery processes defined by CHIME, these were reported negatively or as lacking. Each barrier represented the opposite of the main recovery processes. These barriers are described below.

#### Disconnectedness

Several studies pointed out that many service users had limited social networks, sparse social support and suffered from loneliness and isolation.^20,27,31,34,36^ Many service users felt disconnected from staff and expressed feelings of ‘us and them’ and being socially excluded.^13,22,25,29,33^ Not being respected and not having sufficient or any trust in staff were highlighted as barriers to recovery.^13,29,33^ For example, some experienced that certain staff members would not greet them or that staff members abused their power.^29^ Some service users felt that the strict policies around physical contact limited social interaction.^26,32^ One service user stated: ‘For me mostly it's just missing having that interaction with people’^26^ (p. 945). Furthermore, three papers^14,22,30^ highlighted the dilemma of disclosure, where service users did not trust their relationships with care teams or therapists enough to disclose any difficulties that could hinder a planned leave or activity. One service user stated: ‘(…) I don't think anyone really lets on [about] everything going on in their head’^30^ (p. 238). Another service user reported: ‘Personally, I'd never ask for an appointment with the doctor and say that there is too much noise and too many impressions, and that I, I don't know how to handle it, I might be denied my leave’^14^ (p. 234).

#### Hopelessness

Many FMH service users described how a sense of hopelessness could challenge their recovery. They related the sense of hopelessness to their experience of isolation, segregation and loneliness,^30,36^ and uncertainty about future discharge or length of stay.^14,29,33,36^ For example, one participant in the study by Marklund et al^29^ said: ‘I absconded because of the hopelessness, that I am never getting out of here, regardless of if I behave well year after year, I am still not getting out.’ (p. 239). Furthermore, hopelessness was linked to the lack of involvement and loss of confidence in staff,^29^ being subjected to disempowerment,^35^ never being free from past crimes^21^ and broken promises from staff.^33^

#### Negative identity experience: stigma as an offender

Several papers described how FMH service users experienced stigma as an offender and how this stigma challenged their recovery. Some felt that this stigma was a barrier to their contact with family and friends, whom they feared might be afraid of them.^36^ The experience of being stigmatised had negative effects on their self-esteem.^21^ Many service users were troubled with self-stigma and feelings of guilt.^22,36^ One service user stated: ‘I don't think I'm ever gonna get out of here. And it [is] understandable. I've done some heinous things. But I think I deserve another chance. And it's gonna be really hard for me to get one’^32^ (p. 164). Another service user said: ‘The stories about me as a monster have reached the psychiatric casualty department long before I arrive myself’^31^ (p. 595). The feeling of being constantly tracked and by the possibility of going back to prison caused additional stress.^20^ Furthermore, they assumed their criminal history would hinder future paid work.^26,36^

#### Lack of meaning

A major drawback to recovery was the lack of meaningful activities and meaning in life, particularly on in-patient wards. Some service users reported that their lives on hospital wards felt worthless.^36^ Some reported a poor quality of life: ‘It's not life, it's more of an existence. You just get on with your routine, day in and day out’^26^ (p. 946). Lack of meaning was linked to the lack of meaningful activities,^23^ restrictiveness^26^ and the fact that responsibility was taken away from them.^23^ Boredom was frequently mentioned as a challenge. Being bored was characterised as ‘a long wait’,^14^ having nothing to do,^33^ and a monotonous and repetitive everyday life.^29,36^ Importantly, boredom was seen as a risk factor for aggression and violence,^30^ as well being experienced as harmful for their mental health. Boredom represented a lack of distraction from symptoms. Service users would use sleeping to alleviate boredom, and subsequently stay in bed.^23^ Furthermore, service users were concerned about how they were wasting their time and wasting their lives.^14,29,33^

#### Disempowerment

Finally, more than half of the findings relating to challenges and barriers to recovery concerned disempowerment. Service users felt disempowered by negative staff attitudes or neglect, and by rules and restrictions perceived as punitive or pointless and to which they would adapt with resignation. Some also pointed to the lack of resources, such as staff shortages, as an additional source of disempowerment. Several service users in the studies reviewed highlighted a lack of collaboration in their treatment and care. Instead, they would follow the rules and keep ‘feeding the beast’ – or meeting other people's expectations – just to be able to earn their leave or other rewards.^14,20,28^ One service user stated: ‘You'd better listen to what the staff say and follow their rules as it's their game and not mine so I have to resign myself to these weekly talks’^14^ (p. 235). However, some found that although they had ‘behaved’ and followed their care plan, things did not move forward.^29^ Several service users spoke of loss of freedom and choice in a restricted life, and the feeling of being powerless.^14,22,25,33^ Furthermore, the uncertainty of indefinite time of internment was a great challenge. Some mentioned the lack of clarity in treatment and plans, which counteracted experiencing control in life.^12,25,29^ Others spoke of a lack of a cohesive and united approach to rehabilitation.^22^

### Time and restrictive environments as premises for the forensic recovery journey

The FMH service users characterised their recovery journey as a struggle, and as hard and exhausting work.^14,21,25,30,31^ However, the most prominent finding was the time aspect of the forensic recovery journey, the majority of which was spent in very restrictive environments. Although the long period (‘time’) was seen as an opportunity to change,^25,32^ the FMH service users faced uncertainty because of the long and indefinite time spent in forensic hospitals.^14,20–22,25^ Filling this long period with meaningful activities was seen as an enduring challenge. They also faced inevitable legal and security restrictions while ‘doing time’. Therefore, ‘time’ and ‘restrictions’ may characterise the particular setting or the frame in which the recovery journey can take place for FMH service users, which differs significantly from general psychiatric services. Thus, these are premises that are specific for the forensic recovery journey.

## Discussion

The present paper reviewed 21 studies covering the experiences of personal recovery in FMH service users. Findings suggest that the recovery processes of connectedness, hope, identity, meaning and empowerment, known as the CHIME framework for personal recovery in mental illness, also reflect the recovery processes as experienced by FMH service users. However, this paper has argued that an additional recovery process, relating to feeling safe and being secure, labelled ‘safety and security’, could be added to the CHIME framework to facilitate its extension to the users of FMH services. Safety and security includes experiencing safe care pathways and the active practice of self-management of risk. Hence, the present paper introduces the CHIME-Secure (CHIME-S) framework. Furthermore, findings suggest that the original CHIME categories need some adjustments so that they are better tailored to the FMH population. The original category of connectedness was expanded to include ‘staying connected and being part of a ward community for a long time’, and hope and optimism about the future was expanded to include ‘looking back and looking forward’. Identity was expanded to include ‘identity work: coming to terms with trauma and past offences’, and meaning in life was expanded to include ‘meaningful use of time on the ward and preparing for a meaningful life outside’. Empowerment was expanded to include ‘empowering collaboration within a frame of restrictions’.

Several challenges and barriers for personal recovery for forensic service users have also been identified. All challenges and barriers appeared to represent the opposites of – or the lack of – the recovery processes defined by CHIME, each of which were placed into negatively loaded CHIME categories: disconnectedness, hopelessness, negative identity experience – stigma as an offender, lack of meaning and disempowerment.

### The forensic recovery journey and the forensic recovery processes

The findings in the present review support the idea that the original CHIME categories need some adjustments to fit the forensic population. ‘Time’ and ‘restrictions’ may play a prominent and pervasive role in how we may come to understand the setting – or the frame of – the recovery journey of FMH service users. This understanding affects the translation of the original CHIME recovery processes to cover those of FMH service users. The original category of connectedness was expanded to include ‘staying connected and being part of a ward community for a long time’. Because of the long stays inside restrictive FMH services, and the reduction if not absence of other affirming relationships in the community, the quality of relationships with staff was found to be of particular importance. Moreover, although the ultimate goal is to be successfully integrated into the community, many service users describe the importance in the meantime of being part of a ward community that supports their recovery. This finding is in line with recent work by Aga et al,^38^ who interviewed FMH service users about their experience of connectedness. They found three categories of connectedness: emotional, functional and personal. Emotional connectedness included ‘belonging’ and ‘social inclusion’, whereas functional connectedness included ‘handling time’,^38^ all of which aligns with the new subcategory ‘being a part of a ward community’ and the second-order subcategory ‘support from structures and activities on the ward’ in the CHIME-S framework.

The second category of hope and optimism about the future was expanded to include ‘looking back and looking forward’ as service users; that is, valuing achievements and developing an understanding of their illness and past, and looking forward with a sense of hope. Service users hoped for nothing more than an ordinary life. The original category of identity was understood as a process of identity development, where coming to terms with trauma and past offences was a major part. The category of meaning in life was expanded to include ‘meaningful use of time on the ward and preparing for a meaningful life outside’. This referred to the importance of meaningful activities in their daily lives to fight boredom, symptoms and aggression in addition to learning living skills and skills for coping with difficulties. This finding is in line with previous research that argue that ‘coping with difficulties’ should be included in the CHIME framework.^15,39^

The definition of the empowerment category was expanded to include ‘empowering collaboration within the frame of restrictions’, referring to the need to be involved, heard and taken seriously when planning their treatment and care. Although freedom was the ultimate goal for many, they seemed to accept, as well as aspire to, life-long professional support. It is interesting that their sense of freedom and hope for a normal life did not exclude professional support or medication. Rather, it seemed to be a natural part of their safe planning for the future. This finding is supported by recent literature emphasising the service users’ preference to have ongoing, informal contact with professionals.^38^

The category of safety and security is proposed to be added to the original CHIME framework, to facilitate its extension to the FMH service users. This category refers to feeling safe and being secure, experiencing safe care pathways and the active practice of self-management of risk. Self-management of risk included health-maintaining and risk-reducing strategies and taking responsibility for one's own actions. Nineteen of the 21 papers confirmed the safety and security category. These codes did not fit into any of the existing CHIME categories, and appear to be a specific forensic recovery theme.

A recent study of service user recovery in a forensic step-down rehabilitation unit^8^ supports the findings presented here. They found that forensic residents emphasised mental health management, goal-setting, insight and psychological interventions as important to their recovery, and they appreciated the practicality of preparing for the ‘outside world’.^8^

Importantly, the safety and security issues are based on service users’ needs for safety and their ability to cope with risk, rather than the system's security needs. We may speculate why feeling safe and being secure seem to be particularly relevant for FMH users. One explanation may be that many FMH service users have a history of child abuse,^40–42^ and a significant number suffer from post-traumatic stress disorder.^40^ In a Dutch study by Bohle and de Vogel,^41^ >70% of the participating female FMH service users (*N* = 218) and >60% of the participating male FMH service users (*N* = 218) had experienced at least one type of childhood abuse (emotional or physical abuse or neglect, or sexual abuse).^41^ Furthermore, FMH service users typically describe previous relationships as characterised by feelings of loss, rejection and mistrust.^13^ The significance of this theme may reflect the basic need for safe, high-quality relationships with staff and therapists, as well as the need for protection from hostile environments. Further, it may be specific, as well as essential, for FMH service users to learn how to understand and manage their own risks to reach their goal: to avoid relapse and stay out of hospital. Although rarely explicitly stated by participants, this would also require the safety and security of people around them. Hence, the ultimate goal of many FMH service users and the goal of FMH services may be coincident, and one may be able to have ‘common views of interventions and goals’. The self-management of risk feature of Safety and security could be understood as ‘coping with difficulties’, which, as has been pointed out previously, was missing in the original CHIME framework.^15^

A recovery process is expected to be a measurable dimension of change.^7^ Safety and security as such a measurable dimension of change could include service users feeling safe on the ward and in their relationships with staff, health-maintaining and risk-reducing strategies, and taking responsibility for own actions. This would require involvement in their treatment, knowledge about their illness and understanding of risks (How can I manage my own risk and take responsibility for my own actions?), learning skills to manage difficulties, and support from staff in finding meaningful activities that may prepare them for a life outside FMH services. Insight, or coming out of denial,^34^ may be an initial part of taking responsibility for own actions,^8^ and such insight may emerge from psychoeducation^34^ and understanding of their illness,^21^ and may be helped by support from staff.^33^

The present review supports the findings of two previous reviews from 2016, and expands the conceptual framework for personal recovery in forensic populations. The two previous reviews clearly cover the themes of connectedness, hope and identity. They also support the new subcategories in identity, which refer to work with one's identity,^12^ coming to terms with illness and past,^13^ and coming to terms with having experienced trauma.^12^ Further, they cover safety and security in terms of being protected from a hostile public^13^ and ‘safety and security as a necessary base for the recovery process’.^12^ Although discussed, meaning in life is, to a lesser degree, highlighted as an individual recovery process in the previous reviews. However, 20 of the reviewed papers found that service users called for meaningful activities and meaningful use of time on the wards, as well as support in rebuilding their lives and preparing for a life outside forensic psychiatry. This finding supports the key area for secure recovery ‘meaningful occupation: opportunities for building a life beyond illness’, proposed by Drennan and Woolridge.^11^ Equally, the importance of empowerment is a major finding in the present review, but it is discussed less in the previous reviews. Increasing empowerment for FMH users may be particularly relevant for their recovery, and should be included in a framework intended to guide forensic recovery-oriented practices. This finding supports the idea that FMH service users should be involved in all aspects of their care, through shared decision-making and informed choices, with as much transparency as possible, no matter how limited by circumstances.^11^ Adopting the recovery paradigm in forensic services may increase such involvement.^43^

The present review also supports the findings of the previous reviews that show that recovery processes are closely linked and may overlap to some degree. For example, Shepherd et al identified ‘the dynamics of hope and social networks’, linking hope to connectedness,^12^ whereas Clarke et al^13^ found a strong relationship between connectedness and identity (‘sense of self’). In the present review, we found several papers linking connectedness, meaning in life and empowerment to rebuilding a positive sense of self. Hence, the six recovery processes in the CHIME-S framework may be viewed as categories that depend on and influence each other, and not as entirely separate categories. This is in line with the findings of Whitley and Drake,^44^ who found that recovery categories may overlap, as well as have synergetic interactions.

### The specific challenges and barriers for forensic recovery

One of the most noticeable findings in the present study was the number of challenges and barriers to recovery that were identified in the data, comprising 26% of all findings. Although these findings may be expected from the given premises – the time aspect and the strong restrictive character of forensic ward milieus^45^ – they are important in terms of revealing the weak spots in current recovery-oriented practice as well as the specific challenges for forensic recovery. Although the prolonged time aspect and certain restrictions may be beneficial for personal recovery in some respects, they may also challenge the FMH service users’ opportunities to engage in meaningful relations and activities, hope for the future and autonomy. However, this may also depend on the services’ approach to these challenges. We may question what can be influenced and what is immutable. What we cannot (at least easily) influence is the time aspect, as well as the physical barriers and lack of staff, which could indeed be barriers to personal recovery in FMH services. However, we may influence most challenges and reduce the risk of counteracting personal recovery for FMH service users. For example, the biggest threats to personal recovery in forensic services appear to be disconnectedness and disempowerment. A lot can be done to improve the quality of relationships between service users and staff, and with the task of ensuring that service users have the opportunity to stay connected with their family, friends and peers while detained. Additionally, more efforts could be made to ensure the involvement of service users in discussions about their treatment and defining the clear goals of their care. A recent study has documented successful attempts in increasing service user involvement in secure settings.^43^ Further, staff may carry hope and optimism for the future for service users, as well as support them while they are developing their ‘new identities’.^25^ Staff can value and acknowledge service users’ successes and help them experience an understanding of their illness. Moreover, an effort should be made to ensure that service users are given meaningful activities and opportunities to learn skills to cope with difficulties and everyday life, and to be prepared for a meaningful life outside of FMH services. This includes safety and security issues, such as learning health-maintaining strategies and risk-reducing strategies, as well as overcoming the negative identity experience of stigma as an offender. Many service users found participating in different programmes as particularly helpful in developing new skills, as well as for their personal growth.^28,34,35^

Methodologically, barriers were identified through an overweight of negatively loaded codes within one category. This was the case for the five original CHIME themes, but not for the new emerging theme of safety and security. Following the logic from the other barriers, we may assume that feeling unsafe at the ward or in the relationships with staff almost certainly would be a barrier to personal recovery for FMH service users. Not being able to manage risk, or not learning how to manage risks or relapse in crime, would also challenge personal recovery for FMH service users. Hence, these issues should be given particular attention, to reduce barriers to recovery in FMH services.

All findings taken into consideration, we cannot identify any obvious conflict between forensic recovery-oriented practice and the system's security needs. Although FMH services are concerned about staff and public safety, this should not compromise the personal recovery processes, as stated by service users in the 21 papers reviewed. Actually, there seems to be many ways of optimising personal recovery in FMH services within the frame of security and within the frame of time and restrictions. Quite often, critical voices argue that one cannot apply recovery principles in FMH hospitals. Based on the findings in the present review, the authors will argue that this is not true, and we believe that FMH service users should receive best practice alongside other adult service users in mainstream psychiatric services. The findings of the present study ‘translate’ the personal recovery processes, as defined by the CHIME framework, onto the recovery processes, as described by FMH service users themselves. Thus, these findings may be of particular importance to guide recovery-oriented work in restrictive environments. Furthermore, previous studies suggest that optimising autonomy for service users may reduce aggression and violent incidents in forensic hospitals, as well as in general mental health services.^46–48^

### Study strengths and limitations

The strengths of this study were the systematic review methodology and the multinational and multiprofessional research team, representing two Nordic countries as well as the UK. Nevertheless, this review has several limitations. First, this review only included publications in English language, and it did not include any works from the grey literature. It is possible that the findings reported in such literature could have influenced the findings reported here. However, a substantial number of high-quality, peer-reviewed studies were included, and we have extended rather than contradicted the findings of previous reviews, which adds to the credibility of the present findings. Second, the present review has not analysed any specific phases in the recovery processes, which may vary across services at different security levels and among individuals at different stages along their recovery journey.^7^ For example, there may be considerable differences in patients’ motivation to progress and their readiness for change.^49^ Third, the findings in the present review primarily cover the perception of personal recovery from in-patients. As this may be a strength to identify recovery processes and barriers within FMH hospitals, it may not fully apply to the service users in community low-secure services. Moreover, culture differences, variations across FMH services and differences in legal contexts across countries have not been addressed in this review. This means that some issues in the recovery journey may be more prevalent in specific institutions or countries, which are not acknowledged here. However, the fact that the research group was multinational may have mitigated the impact of this limitation on the study findings.

Finally, a best-fit analysis^37^ would have been an appropriate approach to this study. Although the chosen method in the present study has clear similarities with the best-fit analysis, the thematic synthesis was applied in this study because it enabled line-by-line coding of the data, which the present authors regarded as a comprehensive and exhaustive approach. The best-fit analysis would have been less time-consuming and might have provided different results. This may be a focus for future research.

### Implications for practice and future research

The findings in the present study address the call to tailor the CHIME framework to specific populations,^15^ as well as the need for a framework to guide the recovery-oriented work in FMH services.^4,6^ Although supporting the key ‘secure recovery’ approaches, such as ensuring service user involvement and a collaborative approach to care,^2,11^ the CHIME-S framework may further inform practices to support personal recovery along the care pathway. For example, it suggests an additional focus on supporting the service users’ identity work. Furthermore, it supports an approach to risk assessment and management that is moved from external control toward the service users’ self-management of risk, in which they can be educated to use internal mechanisms to take back control themselves.^11^ Since time and restrictions give premises for the forensic recovery journey, an extra focus must be put on maintaining relationships with family and friends, ensuring meaningful activities throughout the care pathway, the quality of relationships with staff and feelings of belonging to a safe and supportive ward community. Moreover, FMH service providers and management could systematically address each of the challenges and barriers discussed in this paper, to reduce the risk of counteracting FMH service users’ personal recovery.

The CHIME-S framework has the potential to inform the development of a personal recovery measure for FMH service users, which is absent in other forensic recovery tools.^4^ Future research may examine any differences between personal recovery as experienced within the most restrictive environments (i.e. high- and medium-secure services), as opposed to within low-secure services, where access to the community and community integration initiatives can more regularly be found. Furthermore, future research may examine any differences among individuals at different stages along their recovery journey. Finally, future research effort could be put on the optimalisation of the approaches to each of the processes within the frame of time and restrictions.

In conclusion, this systematic review proposes the CHIME-S as a framework for understanding personal recovery in forensic populations. Findings revealed that the expansion of the original CHIME framework is recommended to cover a specific recovery process experienced by FMH service users, which is related to feeling safe and being secure. This includes experiencing safe care pathways and the active practice of self-management of risk. Further adjustments have been proposed in the form of adding new subcategories to the original CHIME categories that are particularly relevant for forensic populations. The CHIME-S framework may guide forensic services in implementing recovery-oriented practices.

## Data Availability

The data that support the findings of this study are available from the corresponding author, M.S., upon reasonable request.

## Supplementary material

For supplementary material accompanying this paper visit http://dx.doi.org/10.1192/bjo.2021.1068. click here to view supplementary material
