# Supplementary material for: Personal recovery and its challenges in forensic mental health: systematic review and thematic synthesis of the qualitative literature
Source: BJPsych Open. 2021 Dec 17;8(1):e17. doi: 10.1192/bjo.2021.1068 (PMC8715254; doi:10.1192/bjo.2021.1068)
Supplement: Supplementary file 1 [file S2056472421010681sup001.docx]

| **CHIME-S (Full version)**  **Recovery processes for forensic patients*** | |
| --- | --- |
| **1 Connectedness** |  |
| **1.1 Peer support and support groups** |  |
| 1.1.1 Availability of peer support |  |
| 1.1.2 Becoming a peer support worker or advocate |  |
| 1.1.3 Supporting others |  |
| **1.2 Relationships** |  |
| 1.2.1 Building upon existing relationships |  |
| 1.2.2 Intimate relationships |  |
| 1.2.3 Establishing new relationships |  |
| 1.2.4 Repairing relationships |  |
| **1.3 Support from others** |  |
| 1.3.1 Support from professionals |  |
| Support over time |  |
| 1.3.2 Supportive people enabling the journey |  |
| 1.3.3 Family support |  |
| 1.3.4 Friends and peer support |  |
| 1.3.5 Active or practical support |  |
| **1.4 Being a part of the community** |  |
| 1.4.1 Contributing and giving back to the community |  |
| 1.4.2 Membership of community organisations |  |
| 1.4.3 Becoming an active citizen |  |
| **1.5 Relationships with staff** |  |
| 1.5.1 Respect |  |
| Accepted as an equal human being |  |
| 1.5.2 Trust |  |
| 1.5.3 Being seen as an individual |  |
| 1.5.4 Staff spending time with patient |  |
| 1.5.5 Quality of therapeutic relationships |  |
| **1.6 Being a part of a ward community** |  |
| 1.6.1 Supportive environment |  |
| 1.6.2 Being supported in spite of drawbacks |  |
| 1.6.3 Support from structure and activities on the ward |  |
| **2 Hope and optimism about the future** |  |
| **2.1 Belief in possibility of recovery** |  |
| 2.1.1 Looking back and looking forward |  |
| **2.2 Motivation to change** |  |
| **2.3 Hope-inspiring relationships** |  |
| 2.3.1 Role-models |  |
| **2.4 Positive thinking and valuing success** |  |
| **2.5 Having dreams and aspirations** |  |
| 2.5.1 Hope for an ordinary life |  |

| **3 Identity** |  |
| --- | --- |
| **3.1 Dimensions of identity** |  |
| 3.1.1 Culturally specific factors |  |
| 3.1.2 Sexual identity |  |
| 3.1.3 Ethnic identity |  |
| 3.1.4 Collectivist notions of identity |  |
| **3.2 Rebuilding/redefining positive sense of self** |  |
| 3.2.1 Self-esteem |  |
| 3.2.2 Acceptance |  |
| 3.2.3 Self-confidence and self-belief |  |
| 3.2.4 Working in with one’s identity |  |
| 3.2.5 Coming to terms with past offences |  |
| 3.2.6 Coming to terms with trauma and having been victimized |  |
| **3.3 Overcoming stigma** |  |
| 3.3.1 Self-stigma |  |
| 3.3.2 Stigma at a societal level |  |
| **4 Meaning in life** | |
| **4.1 Meaning of mental illness experiences** |  |
| 4.1.1 Accepting or normalising the illness |  |
| **4.2 Spirituality (including development of spirituality)** |  |
| **4.3 Quality of life** |  |
| 4.3.1 Well-being |  |
| 4.3.2 Meeting basic needs |  |
| 4.3.3 Paid voluntary work or work related activities |  |
| 4.3.4 Recreational and leisure activities |  |
| 4.3.5 Education |  |
| **4.4 Meaningful social and life goals** |  |
| 4.4.1 Active pursuit of previous or new life or social goals |  |
| 4.4.2 Identification of previous of new life or social goals |  |
| **4.5 Meaningful life and social roles** |  |
| 4.5.1 Active pursuit of previous or new life or social roles |  |
| 4.5.2 Identification of previous of new life or social roles |  |
| **4.6 Rebuilding of life** |  |
| 4.6.1 Resuming with daily activities and daily routine |  |
| 4.6.2 Developing new skills |  |
| 4.6.3 Preparing for life outside forensic hospital |  |
| **4.7 Meaningful use of time** |  |
| 4.7.1 Spending time outside the ward |  |
| 4.7.2 Active use of time |  |
| Reducing boredom |  |
| **5 Empowerment** |  |
| **5.1 Personal responsibility** |  |
| 5.1.1 Self-management |  |
| Coping skills |  |
| Managing symptoms |  |
| Self-help |  |
| Resilience |  |
| Maintaining good physical health and well-being |  |
| 5.1.2 Positive risk-taking |  |
| **5.2 Control over life** |  |
| 5.2.1 Choice |  |
| Knowledge about illness |  |
| Knowledge about treatments |  |
| Knowledge about legal procedures |  |
| 5.2.2 Regaining independence and autonomy |  |
| Regaining freedom |  |
| 5.2.3 Involvement in decision-making |  |
| Care planning |  |
| Crisis planning |  |
| Goal setting |  |
| Strategies for medication |  |
| Medication not whole solution |  |
| 5.2.4 Access to services and interventions |  |
| Tools for recovery |  |
| 5.2.5 Clear goals |  |
| **5.3 Focussing upon strengths** |  |
| **5.4 Mutual collaboration** |  |
| 5.4.1 Common view of means and goals |  |
| 5.4.2 Cooperation and involvement |  |
| **6 Safety and security** | |
| **6.1 Helpful restrictions** |  |
| Dual role of restriction |  |
| Restrictions to gain awareness of need for treatment |  |
| **6.2 Feeling safe and protected on the ward** |  |
| Protection from hostile environment (outside hospital)  Protecting from threats, violence, self-harm and suicide  By presence of staff |  |
| **6.3 Self-management of risk for violence and/or relapse in crime** |  |
| 6.3.1 Taking responsibility for own actions |  |
| 6.3.2 Health maintaining and risk reducing strategies |  |
| **6.4 Safe plans for the future** |  |
| * Lines marked with coloured background are additions to the original CHIME framework defined by Leamy et al., 2011 (7). |  |

| **Specific challenges and barriers in forensic recovery** |
| --- |
| **Challenges and barriers to recovery in forensic psychiatry** |
| **Disconnectedness** |
| Loneliness |
| Not being respected |
| Not having trust in staff |
| Lack of social interaction due to restrictions |
| Dilemma of disclosure |
| **Hopelessness** |
| **Stigma as offender** |
| **Lack of meaning** |
| Boredom |
| Waste of time |
| **Disempowerment** |
| Uncertainty of indefinite time of internment |
| Lack of clarity in treatment and plans |
| Being subjected to disempowerment |
| Feeling subjected to disempowerment |
| By staff attitudes or neglect |
| By rules |
| Limited by rules and restrictions |
| Adapt to rules and care with resignation |
| Lack of collaboration |
| Feeding the beast |
| Loss of freedom |
